# A multi-center blinded study on the efficiency of phenotypic screening methods to detect glycopeptide intermediately susceptible *Staphylococcus aureus *(GISA) and heterogeneous GISA (h-GISA)

**DOI:** 10.1186/1476-0711-6-9

**Published:** 2007-09-24

**Authors:** Andreas Voss, Johan W Mouton, Erika P van Elzakker, Ron G Hendrix, Wil Goessens, Jan A Kluytmans, Paul F Krabbe, Han J de Neeling, Jacobus H Sloos, Nefise Oztoprak, Robin A Howe, Timothy R Walsh

**Affiliations:** 1 Radboud University Nijmegen Medical Centre, Nijmegen University Centre of Infectious Diseases, The Netherlands; 2 Canisius-Wilhelmia Hospital, Department of Medical Microbiology and Infectious Diseases, Nijmegen, The Netherlands; 3 Medical Centre Delft (SSDZ), Department of Medical Microbiology, The Netherlands; 4 Regional Laboratory Twente en Achterhoek, Department of Medical Microbiology, Enschede, The Netherlands; 5 University Medical Centre Erasmus, Department of Medical Microbiology, Rotterdam, The Netherlands; 6 Amphia Ziekenhuis, Department of Medical Microbiology, Breda, The Netherlands; 7 Radboud University Nijmegen Medical Centre, Department of Medical Technology Assessment, The Netherlands; 8 RIVM, National Institute of Public Health, Bilthoven, The Netherlands; 9 Regional Laboratory, Medical Center Alkmaar, Alkmaar, The Netherlands; 10 Zonguldak Karaelmas University Department of Infectious Diseases and Clinical Microbiology, Zonguldak, Turkey; 11 Bristol Centre for Antimicrobial Research & Evaluation, Department of Medical Microbiology, Southmead Hospital, Bristol, UK

## Abstract

**Backgrounds:**

To determine the true incidence of hGISA/GISA and its consequent clinical impact, methods must be defined that will reliably and reproducibly discriminate these resistant phenotypes from vancomycin susceptible *S. aureus *(VSSA).

**Methods:**

This study assessed and compared the ability of eight Dutch laboratories under blinded conditions to discriminate VSSA from hGISA/GISA phenotypes and the intra- and inter-laboratory reproducibility of agar screening plates and the Etest method. A total of 25 blinded and unique strains (10 VSSA, 9 hGISA and 6 GISA) were categorized by the PAP-AUC method and PFGE typed to eliminate clonal duplication. All strains were deliberately added in quadruplets to evaluate intra-laboratory variability and reproducibility of the methods. Strains were tested using three agar screening methods, Brain Heart Infusion agar (BHI) + 6 μg/ml vancomycin, Mueller Hinton agar (MH) + 5 μg/ml vancomycin and MH + 5 μg/ml teicoplanin) and the Etest macromethod using a 2 McFarland inoculum.

**Results and Discussion:**

The ability to detect the hGISA/GISA phenotypes varied significantly between methods and phenotypes. BHI vancomycin and MH vancomycin agar screens lacked the ability to detect hGISA. The MH teicoplanin agar screen was more sensitive but still inferior to Etest that had a sensitivity of 98.5% and 99.5%, for hGISA and GISA, respectively. Intra- and inter-laboratory reproducibility varied between methods with poorest performance seen with BHI vancomycin.

**Conclusion:**

This is the first multi-center blinded study to be undertaken evaluating various methods to detect GISA and hGISA. These data showed that the ability of clinical laboratories to detect GISA and hGISA varied considerably, and that screening plates with vancomycin have a poor performance in detecting hGISA.

## Background

Since the advent of the first glycopeptide intermediately susceptible *S. aureus *(GISA) and its heterogeneous variant (hGISA) in 1997, debate still ensues as to their clinical significance [1-9]. This perhaps has been compounded by the discovery of the vanA-mediated glycopeptide resistant *S. aureus *from the US where the glycopeptide minimum inhibitory concentrations are demonstrably higher and testing issues are also present, but less problematic [10,11]. However, the detection of GISA and hGISA are hampered by the insensitivity of the basic format of standard methods for capturing these phenotypes. Seven years after the publication of Mu50 (GISA) and Mu3 (hGISA), very little has been resolved as to which methods are the most reliable and reproducible. This in part is due to the different definitions ascribed to GISA and hGISA that are subject to methodological differences and variations in (clinical) breakpoints and cut-off values [12,13]. Whilst GISA has a more "homogeneous" resistant population resulting in a higher, stable (thus more reproducible) vancomycin MIC, h-GISA expresses the resistance at approximately 1/10^6 ^of the native population thereby eluding detection by conventional methods. However, strains testing positive by these methods share phenotypic characteristics such as a thickened looser cross-linked cell wall with glycopeptide intermediate *S. aureus *(GISA) [14-16]. Thus, it is possible that hGISA and GISA strains represent the extremes of a common phenotype conferring reduced susceptibility to glycopeptides.

Controlled studies have shown that GISA strains inhibited by a higher vancomycin MIC value are more frequently associated with clinical failures than VSSA. In contrast, the clinical relevance of h-GISA remains controversial. However, numerous case reports have associated hGISA with a poor response to glycopeptide therapy but studies of their clinical significance have been hampered by difficulties in detecting these strains in the diagnostic laboratory and a lack of confirmatory tests. However, a recent observational study comparing the clinical features of bacteraemia due to hGISA (defined by population analysis profiles – area under the curve [PAP-AUC ratio]) and fully vancomycin susceptible MRSA found that hGISA infection was associated with significantly longer time to defervescence (mean 35 vs 2.9 days), and duration of bacteraemia (mean 35 vs. 6.4 days), and a non-significant increase in length of hospital stay (107 vs 37 days) [17]. Clinical failure of vancomycin treatment (defined as fever and bacteraemia >7 days into vancomycin therapy) occurred in 100% (5/5) hGISA cases compared to 2.1% (1/48) MRSA cases.

Studies of the prevalence of GISA and hGISA have also suffered from various non-standardized methods for detection and confirmation of these strains so that inter-country frequencies differ significantly. These differences may reflect genuine geographical variation but are likely to be due to methodological inconsistencies. To facilitate the detection of GISA and in particular hGISA, methods have been proposed where media, inoculum, and period of incubation have been altered [5,18-20]. These include the antibiotic gradient plate, agar screening plates, Etest, population studies (PS) and population analysis profiles – area under the curve assays (PAP-AUC) [21,22]. The most reliable of these is the PAP-AUC; however, it is both specialized and labor intensive. The implementation of these methods for detecting resistance will depend on their intra- and inter-laboratory reproducibility. We describe herein the first multi-center blinded study comparing the reliability of four screening methods in an eight-center setting; namely, to assess and compare the inter-laboratory reproducibility of three "agar screening plates" and the Etest "macromethod" in discriminating VSSA from hGISA/GISA phenotypes.

## Methods

### GISA and h-GISA strains

The study included an international collection of 25 unique strains composed of 10 VSSA, 9 hGISA and 6 GISA as determined by PAP-AUC ratio as previously described [22]. The strains used represented the five major hospital lineages as previously described [6]. All strains were deliberately added in quadruplets to evaluate intra-laboratory variability and reproducibility of the methods. Each of the eight participating laboratories received 100 "numbered" isolates. Investigators were blinded with regard to the isolates' phenotype and were unaware that the collection contained replicates.

### Agar antibiotic screening plates

Laboratories were asked to perform three screening methods. The first utilized Brain Heart Infusion (BHI) (Becton Dickinson, Cockeysville, Md.) agar supplemented with 6 μg/ml of vancomycin (BHI-van) [16]. The other two agar-screening media were prepared in a similar manner as the BHI medium. All media were centrally supplied. They consisted of Mueller Hinton agar (MH- Becton Dickinson, Cockeysville, Md.) with vancomycin (5 μg/ml) (MH-van) and with teicoplanin (5 μg/ml) (MH-teico), respectively [19,23]. Inoculum was prepared as previously described. 10 μl of the inoculum suspension was used for spot inoculation of the agar screening plates. Plates were incubated at 35°C for 18 and 40 hours. Growth was reported after both 24 and 48 hours.

### Etest macromethod

The Etest macromethod for hGISA/GISA testing as recommended by the manufacturer was used as previously described [20]. Essentially, numerous isolated colonies from an overnight agar culture were suspended into MH broth to achieve a turbidity corresponding to 2 McFarland. 100 μl of the inoculum suspension were pipetted onto a standard BHI agar plate and streaked out evenly and then left to dry completely. Etest vancomycin and teicoplanin MIC gradient strips (AB BIODISK, Solna, Sweden) were then applied and the plates incubated at 35°C and read at 24 and 48 hours independently by two technicians. The point of complete inhibition of all growth, including hazes, microcolonies and isolated colonies in the inhibition ellipse, was used as the end-point. Etest macromethod results were interpreted as positive for hGISA/GISA when both vancomycin and teicoplanin modified MICs were ≥ 8 or when the teicoplanin MIC alone was ≥ 12. Etest MIC values that fell in between dilutions e.g. 6 μg/ml were not rounded up.

### Calculation of sensitivity and specificity

All results were entered into SPSS [24]. The sensitivity (= the probability that a "positive" case is correctly classified, thus the false negative rate, and the specificity (= the probability that a "negative" case is correctly classified, or the false positive rate) were calculated. Furthermore, Cohen's kappa was calculated as a measure that expresses the agreement between the evaluations of two raters when both are rating the same object. A value of 1 indicates perfect agreement. A value of 0 indicates that agreement is no better than chance.

## Results and discussion

### Instability of h-GISA phenotype

All GISA and hGISA strains that consistently gave a VSSA phenotype yet were originally categorized as a GISA/hGISA as judged by PAP-AUC (ratio >0.9), were re-sent to the central laboratory to re-confirm their original PAP-AUC profile. Two strains (a Norwegian hGISA and a French hGISA) had lost their ability to express the heterogeneous form of the resistance (PAP-AUC ratios of 0.82 and 0.75, respectively) for vancomycin and were retrospectively excluded from the study. All GISA strains maintained their resistant phenotype.

### Specificity and sensitivity

The sensitivity and specificity of agar screening methods and the Etest macromethod are displayed in table 1. The specificity (i.e. no false positives) of all test methods to define VSSA varied considerably. MH-van and BHI-van gave unacceptable values of 58.7% and 68.4%, respectively. In contrast, both MH-teico and Etest macromethod gave acceptable values of 92.1% and 93.3%, respectively. The ability of each method to detect GISA and hGISA also varied considerably (Table 1). The GISA isolates (as defined by conventional MIC methodology) were virtually all detected by Etest (sensitivity of 99.5%). The agar screening methods varied from one another markedly, the highest sensitivity was MH-teico (95.8%), followed by BHI-van (85.9%) and the lowest was MH-van (50.8%). Predictably, the ability of each method to detect h-GISA varied immensely. Sensitivity values for Etest, MH-teico, BHI-van and MH-van were 98.5%, 85.0%, 4.5% and 1.0%, respectively. Whilst the ability of Etest, and to a lesser degree MH-teico, to detect hGISA maybe regarded as acceptable, the sensitivity values for BHI-van and MH-van are clearly not.

**Table 1:** Sensitivity and specificity of agar screening plates and the Etest macromethod

	correctly identified (%)			
	
	BHI-van	MH-van	MH-teico	Etest
Overall Sensitivity	44.4	25.3	90.3	99.0
Overall Specificity	68.4	58.7	92.1	93.3
h-GISA				
Sensitivity	4.5	1.0	85.0	98.5
GISA				
Sensitivity	85.9	50.5	95.8	99.5

The inter-laboratory reproducibility of the methods as calculated by Cohen's kappa on the quadruplicate samples of each strain is depicted in Table 2. Cohen's kappa values could not be calculated for MH-van for VSSA, MH-van for hGISA and Etest for GISA due to unbalanced variance of components. The inter-laboratory reproducibility for VSSA was acceptable for MH-teico (0.80) and Etest (0.70), and poor for BHI-van (0.30). For h-GISA, BHI-van also performed poorly (0.05) compared to MH-teico (0.93) and Etest (0.88). The reproducibility to detect GISA was generally better, namely 0.70 for BHI-van, 0.91 for MH-van, and 0,89 for MH-teico. The overall intra-laboratory reproducibility was predictably high with values of 0.97, 0.95 and 0.96 for BHI-van, MH-van and MH-teico, respectively.

**Table 2:** Inter-laboratory reproducibility of agar screening plates and Etest macromethod

	BHI-van	MH-van	MH-teico	Etest
VSSA	0,29	*	0,80	0,70
h-GISA	0,05	*	0,93	0,88
GISA	0,70	0,91	0,89	*
All isolates	0,97	0,95	0,96	*

* = unable to estimate due to unbalanced variance components

The marked paucity in controlled studies evaluating the clinical outcome of patients infected with either hGISA or GISA may be due to the difficulty in detecting these resistant phenotypes. Much debate still ensues regarding the clinical significance of GISA and especially hGISA and which methods are best employed to detect them. Methods available vary considerably both at an intra- and inter-country level. For example in the USA, BHI agar screen containing 6 μg/ml of vancomycin is commercially available yet teicoplanin is not available, partly because the drug is not licensed for clinical use in the US. Consequently, most laboratories in the US will preferentially use BHI-van (6 μg/ml) whereas European laboratories will prefer MH-teico (5 μg/ml) or BHI with 4 μg/ml vancomycin [2,23]. Inevitably, the variation in prevalence of hGISA and GISA from these regions may reflect the performance of the methodologies employed rather than the true incidence of the resistance. It is well known that both hGISA and GISA are physiologically different from VSSA, possessing a thicker, less cross-linked cell wall and are usually nutritionally deficient and considerably slower growing [8]. Accordingly, standard antimicrobial susceptibility testing methods are not often appropriate in detecting these phenotypes and studies with richer media, heavier inoculum (to detect the low frequency resistance of 1/10^6^) and extended period of incubation have been documented as alternative testing conditions. However, such non-standardized methods are difficult to control and very few studies have been undertaken examining their inter- and intra-laboratory variability.

This is the first multi-center blinded hGISA/GISA study and has highlighted some of strengths and potential deficiencies within the methodological protocol. For instance, on re-evaluating two strains that gave drastically out-lying results, two hGISA had "lost" their resistance and reverted to VSSA on being transferred from the UK to the Netherlands clearly demonstrating the instability of some strains belonging to the hGISA phenotype. The methods chosen for this study are those that have been proposed by various groups as an initial screen to detect hGISA/GISA. The phenotypic categorization of the strains used in this study was based on PAP-AUC, a specialized method chosen by the referral laboratory that is specifically designed to detect resistant sub-populations that exist with hGISA. Perhaps not surprisingly, the screening methods chosen for this study (agar screening plates and Etest macromethod) varied considerably in their ability to detect hGISA and GISA as judged by the participating laboratories. The poorest performing method under these conditions was MH-van (5 μg/ml) with a sensitivity of 25.3% and a specificity of 58.7%. Whilst, BHI-van (6 μg/ml) performed better, its sensitivity (44.4%) and specificity (68.4%) should be deemed as sub-optimal and caution should be advised when using this screening method. Both Etest macromethod and MH-teico performed very well with a sensitivity of 99.0% and 90.3%, respectively. Although most methods could detect the higher and more homogeneous level of resistance in GISA (apart from MH-van), the biggest variation between the methods arose in detecting hGISA. This in part may be explained by the difference in inoculum and why using a higher inoculum (2 McFarland) proves the rate of detecting the 1/10^6 ^of the cells expressing this type of resistance.

The inter-laboratory variability of these methods varied enormously. The poorest of these was BHI-van that gave a Cohen's kappa value of 0.05 and 0.7 for hGISA and GISA, respectively. Comparatively, the other methods did much better and could be regarded as acceptable. The inter-laboratory variation of a screening method is vitally important if routine microbiology laboratories are going to be encouraged to use such methods. Both MH-teico and Etest performed well, with the latter possessing the highest sensitivity and specificity. Despite the reluctance of some laboratories in implementing the Etest macromethod due to its non-standardized format, the participating laboratories in this study were able to employ it with very few difficulties.

## Conclusion

The debate on the clinical significance of GISA, and in particular hGISA is likely to continue for the foreseeable future [1,6,7]. However, some studies clearly demonstrate that h-GISA is associated with poorer clinical outcome and longer hospitalization [17]. The implementation of non-standardized methods to detect relatively rare and unusual forms of resistance (i.e. GISA and hGISA) must be ratified using multi-center data carried out under blind conditions such as those outlined in this study. The clinical significance of GISA and particularly hGISA can only be truly assessed by first employing appropriate methods to detect it.
